# Enhancing Patient Outcomes in Head and Neck Cancer Radiotherapy: Integration of Electronic Patient-Reported Outcomes and Artificial Intelligence-Driven Oncology Care Using Large Language Models

**DOI:** 10.3390/cancers17142345

**Published:** 2025-07-15

**Authors:** ChihYing Liao, ChinNan Chu, TingChun Lin, TzuYao Chou, MengHsiun Tsai

**Affiliations:** 1 Department of Radiation Oncology, China Medical University Hospital, Taichung City 404, Taiwan; 2 Graduate Institute of Clinical Medical Science, China Medical University, Taichung City 404, Taiwan; 3 Department of Media Design, Tatung University, Taipei City 104, Taiwan; 4 Department of Management Information Systems, National Chung Hsing University, Taichung City 402, Taiwan; 5 A.I. and Data Science Program, National Chung Hsing University, Taichung City 402, Taiwan

**Keywords:** electronic patient-reported outcomes (ePRO), large language model (LLM), retrieval-augmented generation (RAG), head and neck cancer, radiotherapy, weight loss, AI, treatment interruption, patient engagement

## Abstract

Background: Electronic patient-reported outcomes (ePROs) enable real-time symptom monitoring and early intervention in oncology. Large language models (LLMs), when combined with retrieval-augmented generation (RAG), offer scalable Artificial Intelligence (AI)-driven education tailored to individual patient needs. However, few studies have examined the feasibility and clinical impact of integrating ePRO with LLM-RAG feedback during radiotherapy in high-toxicity settings such as head and neck cancer. Methods: This prospective observational study enrolled 42 patients with head and neck cancer undergoing radiotherapy from January to December 2024. Patients completed ePRO entries twice weekly using a web-based platform. Following each entry, an LLM-RAG system (Gemini 1.5-based) generated real-time educational feedback using National Comprehensive Cancer Network (NCCN) guidelines and institutional resources. Primary outcomes included percentage weight loss and treatment interruption days. Statistical analyses included *t*-tests, linear regression, and receiver operating characteristic (ROC) analysis. A threshold of ≥6 ePRO entries was used for subgroup analysis. Results: Patients had a mean age of 53.6 years and submitted an average of 8.0 ePRO entries. Frequent ePRO users (≥6 entries) had significantly less weight loss (4.45% vs. 7.57%, *p* = 0.021) and fewer treatment interruptions (0.67 vs. 2.50 days, *p* = 0.002). Chemotherapy, moderate-to-severe pain, and lower ePRO submission frequency were associated with greater weight loss. ePRO submission frequency was negatively correlated with both weight loss and treatment interruption days. The most commonly reported symptoms were appetite loss, fatigue, and nausea. Conclusions: Integrating LLM-RAG feedback with ePRO systems is feasible and may enhance symptom control, treatment continuity, and patient engagement in head and neck cancer radiotherapy. Further studies are warranted to validate the clinical benefits of AI-supported ePRO platforms in routine care.

## Simple Summary

This study explored the use of an Artificial Intelligence (AI)-powered electronic patient-reported outcomes (ePRO) system combining large language models (LLMs) with retrieval-augmented generation (RAG) to support head and neck cancer patients during radiotherapy. Patients who reported symptoms more frequently experienced less weight loss and fewer treatment interruptions. The system provided real-time, personalized feedback based on reported symptoms, showing promise in improving self-management and care continuity in high-toxicity cancer treatment settings.

## 1. Introduction

Electronic patient-reported outcomes (ePROs) have emerged as a critical tool for symptom monitoring in cancer care, particularly during active treatment [1]. By enabling the real-time collection of patient-reported adverse events and quality-of-life data, ePRO systems empower healthcare providers to detect and manage symptoms promptly, thereby enhancing the efficiency and responsiveness of care [2]. This real-time feedback is especially valuable in high-toxicity treatment regimens such as radiotherapy [3] and chemotherapy [4], where early intervention can mitigate acute symptom exacerbation and reduce avoidable emergency visits or hospitalizations [5].

Concurrently, large language models (LLMs)—such as OpenAI’s ChatGPT and Google’s Gemini—have demonstrated significant potential in healthcare applications, including natural language understanding, patient education, and clinical data interpretation [6]. When integrated with retrieval-augmented generation (RAG) techniques, LLMs can generate responses grounded in validated clinical knowledge, enhancing the professionalism, accuracy, and accessibility of Artificial Intelligence (AI)-generated content for patients [7]. However, few studies have evaluated the integration of LLM-driven real-time education in radiotherapy settings, especially in high-burden cancers such as head and neck cancer [8,9].

Recent evidence has shown that embedding ePRO systems into oncology workflows not only improves patient tolerance of treatment and facilitates early intervention but also reduces hospitalization rates and enhances the quality of clinical decision-making [1,10]. Head and neck cancer patients, in particular, have shown a relatively high willingness to engage with the ePRO platform [11]. Given the frequent occurrence of adverse effects during radiotherapy—such as dysphagia, mucositis, and radiation dermatitis [12]—dynamic symptom monitoring in this population is of particular importance. Nevertheless, in real-world settings with limited clinical resources, interpreting large volumes of ePRO data and providing timely, individualized feedback remains a substantial challenge for healthcare professionals [13,14].

Head and neck cancer patients are especially vulnerable to severe treatment-related toxicities, including mucositis, dysphagia, pain, and taste alterations, which can compromise quality of life, cause significant weight loss, and lead to malnutrition and treatment interruptions [15]. Prior literature has established that weight loss during treatment is closely associated with poor treatment response, decreased local control, and worse overall prognosis [16,17]. While timely, personalized symptom management is essential during radiotherapy, staffing and time constraints often limit the feasibility of providing individualized guidance [18].

Integrating AI tools into ePRO platforms may offer a scalable and interactive solution. In our previous study, we demonstrated the feasibility of applying LLMs to ePRO interpretation [19]. The AI tool exhibited favorable performance in terms of accuracy, completeness, and empathetic tone, and received positive feedback from both patients and healthcare providers. Another study showed that a GPT-4-based application for breast cancer treatment toxicity monitoring demonstrates significant promise in enhancing the quality and efficiency of patient care [20]. These findings suggest that AI-enhanced systems can support communication and reduce the burden on clinical staff [21].

To further improve the specificity and reliability of AI responses, the present study incorporated ePRO with RAG architecture, enabling the LLM to extract tailored guidance and patient education content from curated clinical knowledge databases [7,22].

This work offers three main contributions to the existing literature. First, it presents one of the first real-world applications of an AI-augmented, RAG-enhanced ePRO system in the radiotherapy setting for head and neck cancer patients. Second, it addresses the gap in scalable, individualized symptom management during high-toxicity cancer treatments. Third, it explores the integration of dynamic patient monitoring with AI-driven clinical communication in routine oncology care. The remainder of this article is organized as follows: Section 2 describes the study design and methods, Section 3 presents the results, Section 4 discusses key findings and their implications, and Section 5 concludes with directions for future research.

## 2. Materials and Methods

This study utilized a web-based electronic patient-reported outcome (ePRO) platform developed by Cancell Tech Co., Ltd. (Taipei, Taiwan) [23]. Each patient was required to register an account and complete an intake form with demographic and clinical information, including name, sex, age, cancer type, stage, and current treatment. All patients agreed to the terms of use for the clinical study by signing a consent form, and the data were securely stored on Google Cloud with firewall protection and data encryption protocols.

During radiotherapy, patients were instructed to complete ePRO forms twice weekly. The symptom items were based on adverse event criteria defined by the National Cancer Institute (NCI) and the American Society of Clinical Oncology (ASCO) [1,24]. The data recorded by the patients include quantifiable metrics such as weight and body temperature; scores for quality of life and mood on a scale from 1 to 5, where higher scores indicate better states; and pain scores also ranging from 1 to 5, with higher scores indicating more severe pain. Symptoms such as reduced appetite, stomach discomfort, diarrhea, constipation, nausea and vomiting, coughing, shortness of breath, fatigue, depression, and insomnia are rated on a four-point scale: none for no symptoms; mild, moderate, and severe symptoms; and radiation dermatitis using Common Terminology Criteria for Adverse Events (CTCAE) v5.0 grading, covering a total of 16 symptom items. The ePRO content example is shown as Table 1.

Patients were recruited between January 2024 and December 2024. A total of 42 patients with head and neck cancer were enrolled, all of whom provided written informed consent. The study protocol was approved by the Institutional Review Board of China Medical University Hospital (IRB No.: ePRO_HN_001/CMUH112-REC2-128).

At the initiation of radiotherapy, patients received an onboarding session introducing the ePRO platform, along with paper-based educational handouts on side effect management and nutrition. Radiotherapy was scheduled for 6–7 weeks (30–35 fractions) based on tumor characteristics and treatment guidelines. Patients were expected to complete ePRO entries twice weekly (e.g., Mondays and Thursdays) from the start of radiotherapy to two weeks post-treatment. Missed entries were followed up in the clinic the following day; if not completed within 72 h, they were recorded as missing values.

**Figure 1:**
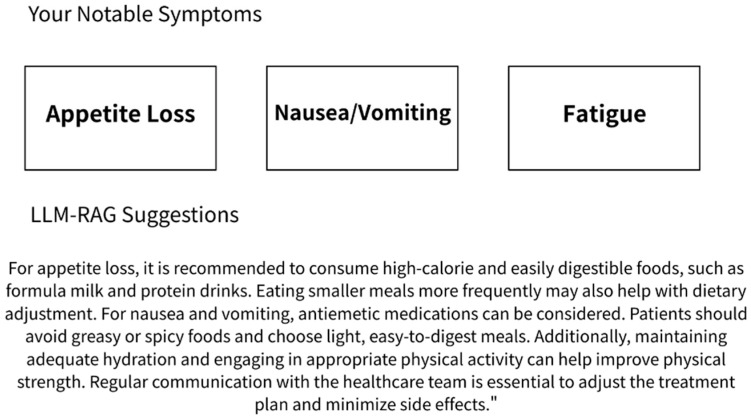
The LLM-RAG feedback example of the nasopharyngeal cancer patient’s ePRO.

After each ePRO submission, a response was automatically generated by a large language model (LLM) system based on Gemini 1.5 and enhanced with a retrieval-augmented generation (RAG) framework. This system delivered real-time, personalized educational feedback based on the patient’s reported symptoms. The content was sourced from the latest National Comprehensive Cancer Network (NCCN) guidelines [25] and an institutional educational database corresponding to the ePRO symptom categories [26]. All AI-generated responses were simultaneously archived in the system backend for clinical review and research analysis. The content had been reviewed in advance by physicians and nursing staff to ensure clinical appropriateness. The LLM RAG feedback example is shown in Figure 1.

**Figure 2:**
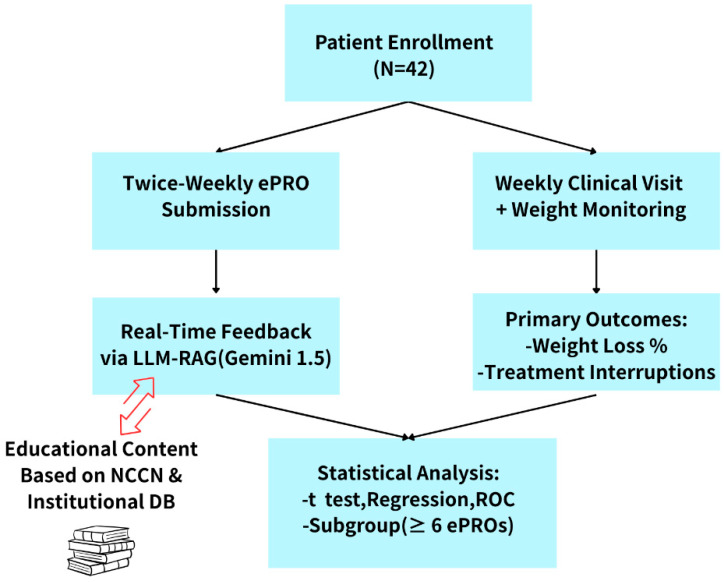
The study workflow. Note: ePRO: electronic patient-reported outcomes, large language model (LLM), retrieval-augmented generation (RAG), National Comprehensive Cancer Network (NCCN).

As part of the routine care pathway, patients attended weekly follow-up visits, during which they underwent physical assessments and weight measurements. Physicians accessed the patients’ ePRO data and LLM-RAG-generated feedback through the backend interface to evaluate treatment response and manage adverse effects. At the conclusion of the study, percentage changes in body weight and the number of radiotherapy interruption days were extracted from the clinical information system and used as the primary clinical outcomes. The study workflow is shown in Figure 2.

Descriptive statistics were used to characterize the overall patient population. Independent-sample t-tests and univariate linear regression were performed to examine the associations between individual clinical variables and the percentage of weight loss, including subgroup comparisons. Pearson correlation analysis was conducted to assess the relationships between weight loss, number of treatment interruption days, and ePRO submission frequency. Variables with *p* < 0.2 in univariate analysis were included in a multivariate linear regression model to identify independent predictors of weight loss. Additionally, a receiver operating characteristic (ROC) curve analysis was performed to determine the optimal ePRO submission threshold for predicting clinically significant weight loss (>5%). A cutoff of six entries was identified as the best discriminative value. All statistical analyses were conducted using SPSS software.

Given the exploratory nature and sample size of this feasibility study, the analysis focused on identifying trends and potential associations. Independent-sample *t*-tests and univariate linear regression were used to assess relationships between patient characteristics, ePRO engagement, and percentage weight loss. Linear regression was selected due to the continuous nature of the primary outcome variable (percentage weight loss) and to allow for adjustment of multiple predictors. Variables with *p* < 0.2 in univariate analyses were entered into a multivariate linear regression model to identify independent predictors. Pearson correlation analysis assessed associations between weight loss, treatment interruption days, and ePRO submission frequency. ROC curve analysis was performed to determine the optimal ePRO submission threshold for predicting clinically significant weight loss (>5%). All analyses were conducted using SPSS (version 18).

Given the relatively small sample size, this study was designed as an exploratory feasibility analysis rather than a hypothesis-driven trial. The sample size was determined by the number of eligible patients during the predefined recruitment period. Therefore, the results should be interpreted as preliminary and hypothesis-generating, with the intention of informing future larger-scale investigations.

## 3. Results

A total of 42 patients with head and neck cancer were enrolled in this study. The cancer subsites included oral cavity (47.6%), nasopharynx (23.8%), and other locations (28.6%). By disease stage, 23.8% were stage III and 47.6% were stage IVA. The cohort was predominantly male (88%), with a mean age of 53.6 years (SD = 11.7) and a mean baseline body mass index (BMI) of 24.1 kg/m^2^ (SD = 4.1).

Regarding treatment, 76.2% of patients received concurrent chemotherapy, and 35.7% had undergone surgical resection. The mean total radiation dose was 6661.9 cGy (SD = 566.5). Over the course of treatment, the average percentage of body weight loss was 5.8% (SD = 4.0), and the mean number of treatment interruption days was 1.5 (range: 0–6). Table 2 summarizes the demographic and clinical characteristics.

From a patient-level perspective (n = 42), the most frequently reported moderate-to-severe symptom was appetite loss (57.1%), followed by fatigue (16.7%) and nausea/vomiting (9.5%). Moderate-to-severe pain was reported in more than half of ePRO entries by 50% of patients. Additionally, 78% of patients reported a quality of life score of moderate or better in over half of their entries, while 38% experienced moderate psychological distress at least once during treatment.

A total of 338 ePRO submissions were collected. The median number of submissions per patient was 8.0 (range: 1–33), with 57% of patients completing at least six entries. Based on a submission-level analysis (n = 338), the most frequently reported moderate-to-severe symptoms were appetite loss (31%), pain (29%), and fatigue (21%). Symptom severity percentages for each item are summarized in Table 3.

Univariate linear regression analyses were conducted to identify factors associated with the percentage of body weight loss during treatment (Table 4). Each β coefficient represents the expected percentage change in weight loss for a one-unit increase in the predictor variable. A positive β value indicates greater weight loss, whereas a negative β value suggests less weight loss or weight preservation.

Factors showing a *p*-value < 0.2 in univariate analysis included receipt of concurrent chemotherapy (β = 2.91, 95% CI: 0.40 to 5.42, *p* = 0.024), fewer than six ePRO submissions (β = 3.12, 95% CI: 0.73 to 5.51, *p* = 0.013), reporting moderate-to-severe pain (score ≥ 3) in more than half of the entries (β = 2.34, 95% CI: −0.15 to 4.83, *p* = 0.065), radiation dose (β = 0.0004 per cGy, 95% CI: −0.0002 to 0.0010, *p* = 0.180), and age (β = 0.08 per year, 95% CI: −0.05 to 0.21, *p* = 0.190). Specifically, regarding BMI, the coefficient (β = 0.143, 95% CI: −0.156 to 0.442, *p* = 0.34) suggests that each 1 kg/m^2^ increase in baseline BMI is associated with a 0.143% increase in weight loss during treatment, although the result is not statistically significant.

As an illustrative example, patients who submitted fewer than six ePRO entries had significantly greater weight loss (β = 3.12, 95% CI: 0.73 to 5.51, *p* = 0.013), indicating that low reporting frequency was associated with a 3.12% higher average weight loss. The 95% CI does not include 0, and the *p*-value is <0.05, supporting the statistical significance of this association.

In the multivariate linear regression model (Table 5), patients receiving concurrent chemotherapy exhibited a trend toward greater weight loss (β = 2.39, 95% CI: −0.44 to 5.22, *p* = 0.095). A trend toward reduced weight loss was observed in those with ≥6 ePRO submissions (β = −1.98, 95% CI: −4.52 to 0.55, *p* = 0.122), although these did not reach statistical significance. Reporting moderate-to-severe pain and higher age also showed trends toward greater weight loss.

To assist interpretation across readers with diverse statistical backgrounds, we have added explanatory notes regarding the meaning of β, 95% CI, and *p*-values in the table legends.

Weight loss was positively correlated with the number of treatment interruption days (r = 0.663, *p* < 0.001) and negatively correlated with ePRO submission frequency (r = −0.396, *p* = 0.0094), suggesting that more frequent reporting may be associated with better symptom control and reduced risk of treatment disruption.

Subgroup analysis compared patients with <6 (n = 18) versus ≥6 (n = 24) ePRO entries. Patients with ≥6 entries experienced significantly less weight loss (4.45% vs. 7.57%, *p* = 0.021) and fewer treatment interruption days (0.67 vs. 2.50 days, *p* = 0.002). Although the proportions of moderate-to-severe pain (37.5% vs. 66.7%, *p* = 0.119) and chemotherapy exposure (70.8% vs. 83.3%, *p* = 0.565) were lower in the high-frequency group, these differences did not reach statistical significance. Overall, frequent ePRO use (≥6 times) was associated with improved symptom management and treatment continuity, supporting the clinical relevance of active patient engagement.

## 4. Discussion

This study is among the first to explore the integration of an ePRO system with an LLM enhanced by RAG in the context of head and neck cancer radiotherapy. The proposed AI-supported system serves a dual function: first, to assist clinicians in dynamically monitoring and visualizing patient-reported symptoms over time; and second, to provide timely, personalized, AI-generated response education to patients based on their reported symptoms. Our findings demonstrate that this approach is clinically feasible and may improve patient engagement, symptom management, and overall care outcomes during the radiotherapy treatment course.

The results are consistent with the existing literature, such as the study by Basch et al. [1], which demonstrated that ePRO use reduces emergency department visits and hospitalizations among cancer patients [27]. In our cohort, no patients required emergency care or hospitalization. Patients who submitted six or more ePRO entries experienced significantly less weight loss and fewer treatment interruptions, indicating a positive correlation between higher engagement and improved treatment tolerance—an outcome previously linked to survival benefits in cancer populations with immunotherapy [28].

Several prior studies have evaluated the implementation of ePRO systems in oncology settings, including head and neck cancer. Katzel et al. found that ePROs can be effectively integrated within electronic medical record (EMR) systems to facilitate symptom monitoring during radiotherapy [3]. Zebralla et al. demonstrated that ePRO-based follow-up using the OncoFunction platform was feasible, improved data acquisition, and enhanced visualization of patient outcomes [29]. However, these systems primarily relied on manual clinician responses to ePRO alerts. In contrast, our system introduces an AI-enhanced approach in which patient-reported symptoms automatically trigger real-time, personalized educational feedback via LLM-RAG. This innovation supports scalability and patient self-management, reducing reliance on immediate clinical intervention. To our knowledge, this is the first real-world implementation of such a model in the radiotherapy setting.

Consistent with prior literature [30], chemotherapy remained a key predictor of weight loss in our study. Symptoms such as appetite loss, fatigue, nausea, vomiting, and pain were frequently reported and are well-known contributors to nutritional decline. Although not statistically significant, both age and total radiation dose showed trends toward greater weight loss, suggesting that older patients and those receiving high-dose treatment may experience a heavier symptom burden [31,32]. These findings highlight the potential utility of AI-enhanced ePRO systems in proactively supporting vulnerable subgroups through personalized symptom education and early alerts.

In our data, patients reporting persistent moderate-to-severe pain tended to lose more weight, while those who submitted more ePRO entries reported less pain overall. Although psychological distress and emotional symptoms were not directly correlated with weight loss or treatment interruption, nearly 40% of patients experienced moderate distress. Prior studies have shown associations among pain [33], distress [34], treatment disruption [35], and survival in head and neck cancer. These findings underscore the value of the ePRO-LLM system in detecting both physical and psychological symptoms early and delivering supportive content tailored to the outpatient setting [36,37].

While higher submission frequency was associated with better outcomes, we acknowledge potential bias due to unmeasured patient factors. All patients in this study received the same routine outpatient clinical care, yet those who submitted more ePRO entries still had better outcomes. This suggests the observed differences may stem from greater patient engagement [38,39], rather than more intensive clinical intervention. However, patients with lower reporting frequencies may have been too unwell or unmotivated to submit entries, leading to under-reporting or missed detection of adverse events. This potential bias highlights the importance of considering patient condition and engagement when interpreting ePRO data.

Those who submitted fewer ePRO entries (e.g., <6 submissions) may have underreported or unrecognized symptoms, potentially leading to reduced detection rates of certain adverse events. To improve adherence and minimize missing data, future systems could incorporate chat-enabled ePRO platforms [21], voice-assisted technologies (e.g., Apple Siri [40]) or proactive reminders from caregivers to improve adherence and reduce missing data.

This study has several limitations. First, it was a single-center, single-arm, exploratory feasibility study with a relatively small sample size. Second, the absence of a control group without AI-based education limits our ability to isolate and evaluate the independent clinical effect of the LLM-RAG system. Third, the educational content generated by the LLM-RAG was reviewed for appropriateness by clinical experts prior to implementation and shown in previous studies to have high accuracy in simulating multidisciplinary roles [19]. This study did not track the accuracy of individual AI responses, the patient adoption rate, or downstream behavioral actions. Fourth, patients who completed fewer than six ePRO entries may have contributed less reliable symptom data, potentially leading to underestimation of adverse event frequencies and reduced representativeness of symptom burden across the cohort. Fifth, detailed baseline nutritional status and supportive care interventions (e.g., individualized dietary counseling, enteral nutrition use) were not systematically collected, limiting our ability to control for confounding factors that may influence patient-reported outcomes. Therefore, while the findings suggest potential benefits associated with AI-triggered feedback, the causal relationship between AI-generated content and clinical outcomes remains indirect. Additionally, a gap may exist between patient-facing AI suggestions and actual clinical decision-making.

Nevertheless, the system’s automation and real-time feedback significantly improved the accessibility and responsiveness of patient education. It was well accepted by patients and shows strong potential for integration into clinical care. Future work should implement AI response pre-screening, evaluate patient adherence, and conduct effectiveness tracking. Randomized controlled or prospective studies with comparison groups are warranted to further validate this system across cancer types and treatment settings.

## 5. Conclusions

In conclusion, the integration of ePRO with LLM-RAG educational feedback in the radiation oncology setting demonstrates preliminary feasibility and acceptability. This approach shows promise in supporting early and comprehensive symptom detection, timely intervention, and extended self-management by patients. Our findings reinforce the clinical potential of combining real-time digital monitoring with AI-generated education to enhance patient engagement, improve symptom tracking, and reduce treatment risks—laying the groundwork for novel supportive care models in high-toxicity cancer settings such as head and neck radiotherapy. Broader implementation into routine clinical practice is encouraged to help optimize care quality and treatment continuity.

## Figures and Tables

**Table 1:** Example of ePRO data from a nasopharyngeal cancer during radiotherapy.

Date	Weight (kg)	Body Temperature (°C)	Quality of Life (The Higher, the Better)	Pain Score (The Higher, the More Painful)	Mood (The Higher, the Better)	Appetite Loss	Epigastric Discomfort	Diarrhea	Constipation	Nausea/ Vomiting	Cough	Dyspnea	Fatigue	Depression	Insomnia	Radiation Dermatitis (CTCAE v5.0)
9 January 2024	90	36.9	3	3	3	Mild	Moderate	None	Moderate	Mild	None	None	Moderate	Mild	Moderate	None
16 January 2024	90	36.9	3	2	3	Mild	Moderate	None	Mild	Mild	None	Mild	Mild	Mild	Mild	None
19 January 2024	90	36.8	2	3	2	Moderate	Mild	None	None	Moderate	Mild	Mild	Moderate	Mild	Mild	None
23 January 2024	89	36.7	3	2	4	Mild	Mild	None	None	Mild	Mild	Mild	None	None	None	Grade 1
26 January 2024	88	36.8	3	2	3	Moderate	Mild	None	Moderate	Mild	Mild	Mild	Mild	None	None	Grade 1
30 January 2024	88	36.8	2	2	2	Moderate	Mild	None	Moderate	Moderate	None	Mild	Mild	Mild	None	Grade 1
2 February 2024	87	36.7	3	2	3	Moderate	Mild	None	Moderate	Moderate	Mild	Mild	Mild	Mild	Mild	Grade 1
6 February 2024	87	36.8	2	2	2	Moderate	Mild	None	Moderate	Moderate	Moderate	Mild	Mild	Mild	Mild	Grade 1
13 February 2024	86	36.9	3	2	3	Moderate	Mild	None	None	Mild	Mild	Mild	Mild	Mild	None	Grade 1

Notes: Common Terminology Criteria for Adverse Events (CTCAE).

**Table 2:** Patient characteristics.

Characteristics	Numbers
Gender: Male vs. Female	37 vs. 5
Nasopharynx Cancer	10
Oropharynx Cancer	7
Oral Cavity Cancer	20
Hypopharynx Cancer	2
Larynx Cancer	1
Unknown Primary Cancer	1
Age (mean)	53.6 (30–83)
Stage I:II:III:IVA	7:5:10:20
Received Operation	15
Received Concurrent Chemotherapy	32
Radiation dose (mean)	6661 cGy (4600–7000 cGy)
Body Mass Index (mean) kg/m^2^	24.1 (15–33)
ePRO Submissions Times < 6 vs. ≥6	18 vs. 24
Half Time Experienced ≥ Moderate Pain	21 of 42
Half Time Experienced Good QOL	33 of 42
Ever Experienced Moderate Distress (Bad Mood)	16 of 42
Body Weight Loss % (mean)	5.8% (0–18.3)
Radiotherapy Interruption Days	1.5 (0–6)

Notes: ePRO: electronic patient-reported outcomes, QOL: quality of life.

**Table 3:** Percentage of moderate or higher symptom severity reported in ePRO submissions.

	Quality of Life ≤ 2 (The Higher, the Better)	Pain Score ≥ 3 (The Higher, the More Painful)	Mood ≤ 2 (The Higher, the Better)	Appetite Loss ≥ Moderate	Epigastric Discomfort ≥ Moderate	Diarrhea ≥ Moderate	Constipation ≥ Moderate	Nausea/ Vomiting ≥ Moderate	Cough ≥ Moderate	Dyspnea ≥ Moderate	Fatigue ≥ Moderate	Depression ≥ Moderate	Insomnia ≥ Moderate	Radiation Dermatitis (CTCAE v5.0) ≥ Gr.2
% of ePROs	20	29	20	31	8	3	9	11	4.7	2	21	12	3.5	2

Notes: ePRO: electronic patient-reported outcomes, CTCAE: Common Terminology Criteria for Adverse Events.

**Table 4:** Univariate linear regression analysis of factors associated with percentage weight loss during radiotherapy.

Factors	β	95% CI	*p*-Value
Sex	0.57	−3.368–4.508	0.7713
Age	0.071	−0.036–0.178	0.1898
Stage	0.555	−0.563–1.674	0.3217
Surgery	−1.464	−4.077–1.148	0.264
Chemotherapy	3.239	0.439–6.04	0.0245 *
Radiation Dose	0.002	−0.001–0.004	0.1769
Body Mass Index	0.143	−0.156–0.442	0.34
ePRO ≥ 6 times	−3.054	−5.431–0.677	0.0131 *
Pain	2.281	−0.156–4.718	0.0658
Quality of Life	−1.811	−4.856–1.234	0.2364
Distress (Bad Mood)	0.585	−2.027–3.197	0.6535

Notes: β represents the estimated percentage change in body weight loss associated with a one-unit increase in the predictor variable. A positive β indicates greater weight loss, while a negative β indicates less weight loss or weight preservation. CI = confidence interval. ePRO: electronic patient-reported outcomes.*: *p* < 0.05.

**Table 5:** Multivariate linear regression model for predictors of percentage weight loss during radiotherapy.

Factors	β	95% CI	*p*-Value
Chemotherapy	2.3931	−0.4374–5.2235	0.095
ePRO ≥ 6 times	−1.9845	−4.5233–0.5543	0.1216
Pain	1.8345	−0.4584–4.1275	0.1134
Age	0.0785	−0.0193–0.1763	0.1122
Radiation Dose	0.0017	−0.0004–0.0037	0.1122

Notes: β represents the estimated percentage change in body weight loss associated with a one-unit increase in the predictor variable. Positive β values indicate greater weight loss; negative β values suggest less weight loss. CI = confidence interval. ePRO: electronic patient-reported outcomes.

## Data Availability

The study’s detailed data are unavailable due to privacy or ethical restrictions.
